# Hepatitis C community prevalence is over-estimated: a prospective, birth cohort study

**DOI:** 10.1007/s11845-023-03604-2

**Published:** 2024-01-29

**Authors:** P. Aiden McCormick, Marie O’Grady, Cillian F. De Gascun, John S. Lambert, Orla Crosbie, Susan McKiernan, Maeve Skelly, Paul Holder, Garry Courtney, Brian Hennessy, Kevin Walsh, Roisin Twohig, Kate Browne, Tessa O’Gorman, Vivion Crowley, Seán J. Costelloe, Roz O’Byrne, Elizabeth Whitney, Orla Gildea, Noreen Montgomery

**Affiliations:** 1 National Hepatitis C Treatment Program HSE, Liver Unit, St. Vincent’s University Hospital and UCD, Elm Park, Donnybrook, Dublin 4, DO4 T6F4 Ireland; 2 National Virus Reference Laboratory, UCD, Dublin, Ireland; 3 Mater and Rotunda Hospitals and UCD, Dublin, Ireland; 4 Cork University Hospital and UCC, Cork, Ireland; 5 St. James’s Hospital, Dublin, Ireland; 6 Dip Qi University Hospital Limerick, Limerick, Ireland; 7 St. Luke’s Hospital, Kilkenny, Ireland; 8 University Hospital Waterford, Waterford, Ireland; 9 Sligo University Hospital, Sligo, Ireland; 10 Mater Misericordiae University Hospital, Dublin, Ireland; 11 St. James’s University Hospital, Dublin, Ireland; 12 University Hospital Cork, Cork, Ireland; 13 University Hospital Limerick, Limerick, Ireland

**Keywords:** Birth cohort, Cirrhosis, Direct acting antiviral agents DAA’s, Epidemiology, Hepatitis C

## Abstract

**Background:**

Hepatitis C virus infection is often asymptomatic, and many patients may be unaware they are infected. Community-based, birth cohort screening has been advocated to identify these patients. It has been estimated that 0.7–1% of individuals born between 1965 and 1985 in Ireland are infected. The cost-effectiveness of screening is critically dependent on the population prevalence.

**Aims:**

The aim is to determine the community prevalence of hepatitis C virus infection in the birth cohort 1965–1985.

**Methods:**

Residual serum samples from blood tests ordered by community general practitioners were anonymised and analysed for the presence of hepatitis C antibody ± antigen. Twelve large general hospitals throughout the country participated.

**Results:**

A total of 14,320 samples were tested, 9347 of which were from the birth cohort 1965–1985. Seventy-two samples were positive for hepatitis C antibody of which 12 were positive for hepatitis C antigen (17%). The overall prevalence of hepatitis C antigen in the birth cohort was 0.09%. A higher prevalence (0.39%) was identified in males in two urban areas of Dublin.

**Conclusions:**

Hepatitis C virus seroprevalence was much lower than previously estimated. The proportion of antibody positive patients with hepatitis C antigen was also lower than expected suggesting the effects of treatment and/or high spontaneous viral clearance. Universal birth cohort screening is unlikely to be cost-effective. Targeted birth cohort screening in high prevalence areas could be considered.

The world health organization (WHO) has set out elimination targets for hepatitis C to be achieved by 2030 [1]. The targets state that 90% of patients with chronic hepatitis C should be diagnosed and 80% of those diagnosed, treated, by 2030 [2]. The difficulty with assessing progress towards these targets is that they require robust data on population prevalence. Two studies have provided population estimates for Ireland. The first is by Thornton et al., estimated there were between 19,826 and 49,565 individuals with chronic hepatitis C in Ireland in 2009 [3]. This study was based on public health notification data and assumed that 75% of infections became chronic. Garvey et al. looked at stored sera from the National Virus Reference Laboratory, excluding samples from high-risk settings and estimated there were 19,606 chronically infected adults in 2016 [4]. Public health notification data suggests that 70% of chronically hepatitis C infected patients were born between 1965 and 1985 [5]. Based on these data, the Irish Health Information and Quality Authority (HIQA) estimated the prevalence of hepatitis C infection in the birth cohort 1965–1985 was approximately 0.8%. For individuals aged 46–50 and 51–56 years old in 2021, estimated prevalence was 1.14% and 1% respectively [6].

The Irish national hepatitis C screening guidelines recommended that a screening program for this birth cohort be considered [7]. A health technology assessment concluded that this approach would be cost-effective, given the estimated prevalence [6]. The estimated cost of an opportunistic birth cohort screening program was €44 m over 5 years. A systematic screening program would cost approximately €65 m. In view of the uncertainty around prevalence estimates, HIQA recommended further research and a possible pilot program. The aim of this study was to estimate the prevalence of chronic hepatitis C virus infection, particularly in the birth cohort 1965–1985. Given that the proposed screening program would be based in primary care, we tested anonymised, residual samples from routine blood tests ordered by general practitioners in a range of hospitals throughout the country. Two studies were performed. The first looked at birth cohort samples alone. The second looked at all adult samples.

## Methods

Two studies were performed. The first study looked at residual serum samples from general practitioner requested blood tests in eight hospitals, three of which were in Dublin. These hospitals included St Vincent’s University Hospital, Mater Misericordiae University Hospital, St James’s University Hospital, Cork University Hospital, University Hospital Limerick, University Hospital Waterford, St Luke’s Hospital Kilkenny and Sligo University Hospital. All patients were in the birth cohort group, and the study included equivalent numbers of males and females. Residual samples were anonymised, batched and sent to the National Virus Reference Laboratory in UCD for analysis. The only information retained on the samples was the sex of the patient. Ethical permission was obtained from the research committees in St Vincent’s University Hospital, Mater Misericordiae University Hospital, St James’s Hospital, University Hospital Limerick, Cork University Hospital and University Hospital Waterford. Sligo University Hospital and St Lukes’s Hospital Kilkenny accepted the ethics approval from St Vincent’s University Hospital.

The second study was performed using residual sera, provided by the National Serosurveillance Programme (NSP) following approval from its National Steering Committee. This was left over sera, following initial residual testing for SARS-CoV-2. The NSP conducts systematic sampling and analysis of anonymised residual left over blood samples, originally collected for clinical testing, which are due to be discarded. A network of participating acute-hospital clinical chemistry laboratories: Letterkenny University Hospital, St. Vincent’s University Hospital, University Hospital Limerick, Galway University Hospital, Beaumont Hospital and Tallaght University Hospital provided the residual specimens sourced from primary care which were then analysed in the National Virus Reference Laboratory. Sampling occurs in approximately 6 weekly cycles. Sample numbers in age groups are selected for inclusion based on the 2021 estimates of the Irish national age distribution of the Irish national population. Each site provides between 100 and 300 specimens per cycle. All data held or stored by the NSP are anonymous and not personally identifiable. As seroprevalence is a core surveillance activity for which the Health Protection Surveillance Centre is legally mandated, no individual patient consent is required. The samples sourced from the sero-surveillance programme were from individuals over 18 years old, reflecting the national age distribution of the Irish population and not restricted to the birth cohort (1965–1985) range.

All assays were performed at the National Virus Reference Laboratory, UCD Belfield, Dublin. HCV antibody status was determined using the HCV Ab Architect Abbott HCV antibody test (Abbott Diagnostics, Wiesbaden, Germany). Specimens exceeding the manufacturer’s cut-off of 1.0 were tested for hepatitis C virus antigen using the HCV Ag Architect Abbott HCV antigen test. Samples that proved reactive in the anti-HCV assay but negative for HCV Ag were further investigated using the Fujirebio Innotest Ab IV assay (Fujirebio Europe N.V., Gent, Belgium) and/or Bio-Rad Monolisa anti-HCV Plus vs 3.0 (Bio-Rad, Marnes-la-Coquette, France) assays. Persistently reactive anti-HCV samples were then tested using the Fujirebio INNO-LIA HCV score line immunoassay (Fujirebio Europe N.V., Gent, Belgium) to determine the true anti-HCV status of the sample. Because of the size of the sample, the raw data is presented, no assumptions are made and no statistical testing or manipulation was carried out.

## Results

**Fig. 1:**
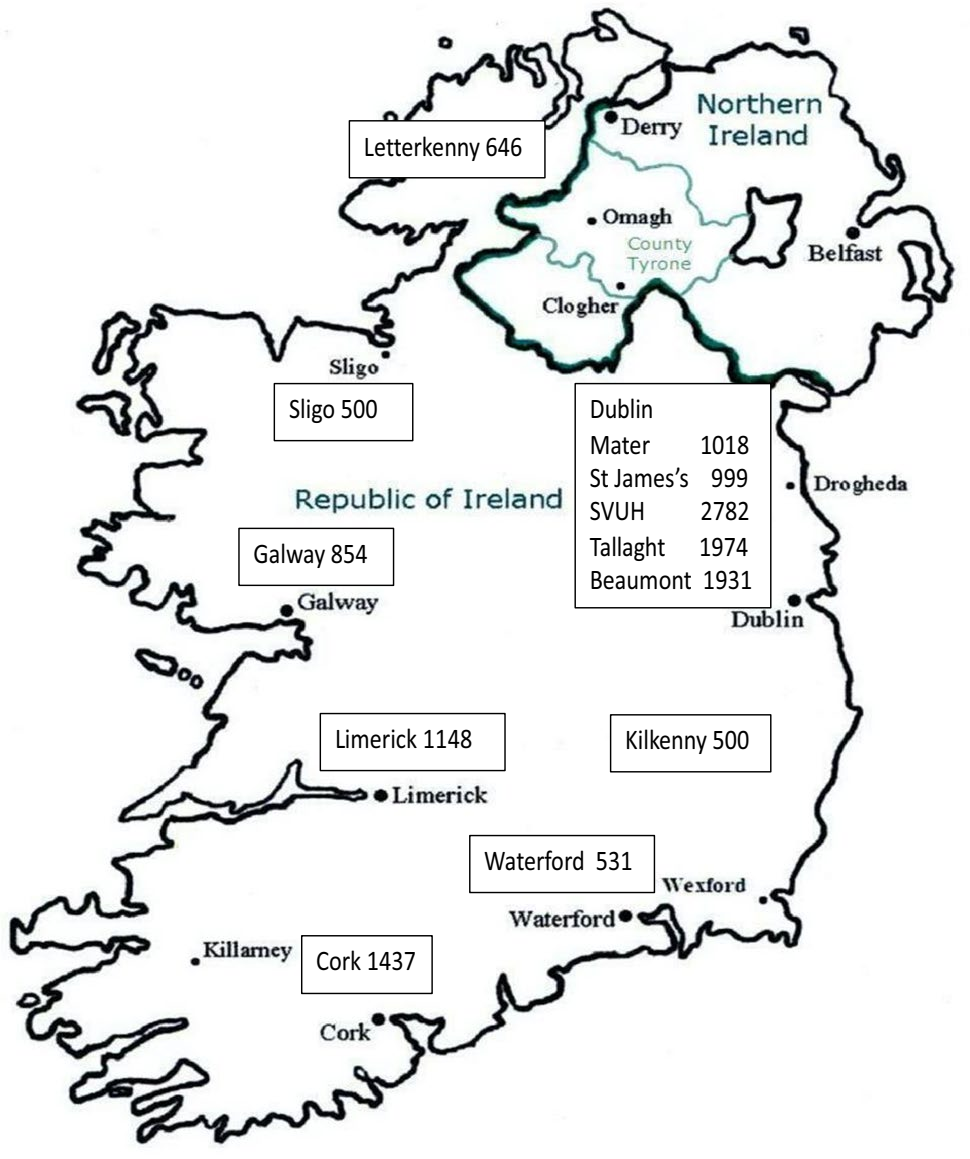
Geographical distribution of hospitals and the number of samples provided for the studies. SVUH, St Vincent’s University Hospital

A total of 6080 samples were tested in the initial birth cohort study and an additional 8240 in the second study. The birth cohort samples were collected during 2021 and the first half of 2022. The second study samples were collected in the second half of 2022 and the first quarter of 2023. Twelve hospitals contributed samples, including all five major Dublin hospitals. The contributing hospitals and the number of samples received from each hospital are summarised in Fig. 1. A total of 14,320 samples were tested. This included 7096 males, 6944 females and 280 samples where the sex was not recorded.

Seventy-two samples were hepatitis C antibody positive. The results are summarised in Table 1. Twelve of the 72 antibody samples (17%) were hepatitis C antigen positive. There were a total of 63 initially equivocal or weakly positive samples which were considered to be negative after confirmatory testing. In the initial birth cohort study, two samples were hepatitis C antigen positive, one male and one female. In the second study, 10 samples were antigen positive, eight males and two females. Six of the 10 were in the birth cohort age group (four males, two females), one male was younger and three males were older. Nine of the antigen positive patients were from Dublin, two from Cork and one from Limerick. All the antigen-positive patients in Dublin came from Tallaght University Hospital (5) and Beaumont Hospitals (4). These hospitals are situated in South West and North East Dublin respectively. Hepatitis C antigen prevalence in the various groups is shown in Table 2. The overall prevalence in this population is low at approximately 0.1%. There appears to be a higher prevalence in North East and South West Dublin. In males, this is 0.39%, and in males and females combined, it is 0.33%.

**Table 1:** Detection of hepatitis C antibody and antigen by age and sex distribution for individuals born before, during or after the birth cohort years 1965–1985

	**Born before 1965**		**Born between 1965 and 1985**			**Born after 1985**	
	Male	Female	Male	Female	Not recorded	Male	Female
**Numbers**	1213	1242	4632	4435	280	1251	1267
**Hepatitis C Antibody positive**	7	0	41	14	0	8	2
**Hepatitis C antigen positive**	3	0	5	3	0	1	0

**Table 2:** Prevalence of hepatitis C antigen in the two studies combined + various sub-groups. Birth cohort refers to those individuals born between 1965 and 1985 inclusive

	Prevalence	Prevalence %
Total sample	12/14,320	0.08%
Birth cohort	8/9347	0.09%
Non-birth cohort	4/4973	0.08%
Male birth cohort	5/4632	0.11%
Birth cohort: Cork	2/1203	0.17%
Birth cohort NE + SW Dublin	5/1510	0.33%
Male birth cohort NE + SW Dublin	3/779	0.39%

## Conclusion

On the basis of our studies, the prevalence of hepatitis C virus infection in the general adult population in Ireland appears to be low at less than 0.1%. The national prevalence in the birth cohort 1965–1985 is also low. In males, it appears to be about 0.11%. A higher prevalence was identified in North East and South West Dublin. There, the birth cohort prevalence was 0.33%, and in males, it was 0.39%. The HIQA health technology assessment estimated that birth cohort screening for hepatitis C would be cost effective at a prevalence of approximately 0.4% or higher. On the basis of these results, national birth cohort screening is unlikely to be cost-effective. However, birth cohort screening targeted at higher prevalence areas in Dublin may be cost-effective.

Given these results, we estimate that there are between 3500 and 5000 patients with active chronic hepatitis C virus infection in Ireland. An interesting finding in this study was that only 17% of patients found to be antibody positive were positive for hepatitis C antigen. This compares to the assumption that 75% of hepatitis C virus infections become chronic in the Thornton study [3, 8]. This suggests that a large proportion of infected patients either spontaneously cleared the virus or had effective curative antiviral therapy. If this is the case, then between 20,000 and 30,000 individuals may have been infected with the virus at some time in the past. This would be in line with previous estimates.

Thornton et al. published the first systematic estimates of hepatitis C prevalence in Ireland in 2011 [3]. They used data from diagnostic samples in the National Virus Reference Laboratory (1989–2004) and national notification data from 2004 to 2009. Hepatitis C became a notifiable disease in Ireland in 2004. They estimated the true number of chronically infected individuals ranged from 19,826 to 49,565. These figures presumed under-diagnosis rates of 50 to 80%. The estimates also assumed that 75% of hepatitis C virus infections became chronic, i.e. 75% of individuals with hepatitis C antibodies had chronic hepatitis C virus infection. Up till 2012, patients with hepatitis C antibody positivity were notified as chronically infected. The combination of these factors may have led to an over-estimate of disease prevalence. Garvey et al. tested residual anonymised sera in the national virus reference laboratory in 2016. These samples were tested for other viruses and stored for 2 years before being scheduled for disposal. They thus represented samples from before the DAA era. Thirty-three individuals were HCV antigen and antibody positive, and the estimated national prevalence was 19,606 chronically infected individuals. The weighted prevalence in the birth cohort was approximately 1%. This is far higher than our results. A possible reason for this difference could be that this study pre-dated the DAA era where highly effective curative treatment became widely available. In addition, it was a retrospective study looking at samples that had been sent to the virus reference laboratory for other viral tests. While stringent efforts were made to exclude individuals with risk factors, this was a different selection process from our study, which used residual sera from routine biochemistry requests from general practitioners.

The strengths of this study are that it is prospective, community based, has a large sample size and a wide geographical coverage. The samples were blood tests ordered by general practitioners and therefore are more likely to be representative of the general population than hospital-based studies. The major weakness is that some high-risk populations are probably under-represented. These include actively drug-using populations, homeless persons, migrant populations or individuals in prison. On the other hand, all of these populations already have specific services targeting blood-borne virus screening and targeted treatment programs. The aim of this study was to determine whether there was a large reservoir of un-diagnosed chronic hepatitis C virus infection in the community, particularly among individuals who may be unaware if they are infected. Our results suggest there is a significant un-diagnosed cohort, but the numbers are much lower than previously predicted.

In summary, our results suggest that the prevalence of chronic hepatitis C virus infection in the Irish adults is approximately 0.1%, which is much lower than previously estimated. The fact that only 17% of hepatitis C antibody–positive patients had hepatitis C antigen suggests that spontaneous clearance rates are high, or there has been a significant uptake of curative antiviral treatment or a combination of both. These results also suggest that Ireland is on track to achieve the WHO elimination targets for hepatitis C.

## Data Availability

Data may be made available to academic researchers on request.
